# Cononsolvency‐Assisted Fabrication of Hydrogels and Microgels With Tunable Porosity, Diffusivity and Stiffness

**DOI:** 10.1002/marc.70423

**Published:** 2026-09-05

**Authors:** Maria I. Pieper, Daniel D. Stöbener, Bernhard Häßel, Richard Hoogenboom, Andrij Pich

**Affiliations:** ^1^ Institute of Technical and Macromolecular Chemistry RWTH Aachen University Aachen Germany; ^2^ DWI‐Leibniz Institute for Interactive Materials e.V. Aachen Germany; ^3^ Supramolecular Chemistry Group, Centre of Macromolecular Chemistry (CMaC), Department of Organic and Macromolecular Chemistry Ghent University Ghent Belgium; ^4^ Aachen Maastricht Institute for Biobased Materials (AMIBM) Maastricht University, Brightlands Chemelot Geleen The Netherlands

**Keywords:** cononsolvency, droplet‐based microfluidics, hydrogels, microgels, porosity, responsiveness

## Abstract

Porous materials including hydrogels offer high potential for applications requiring controlled mass transport, cell infiltration, and mechanical properties, such as tissue engineering, drug delivery, or soft robotics. We report a facile method for the synthesis of hydrogels and microgels with tunable porosity, offering control over diffusivity and mechanical properties. By exploiting the cononsolvency of polymers based on dimethylacrylamide (DMAAm) and hydroxyethylacrylamide (HEAAm) in water‐acetonitrile mixtures, we produce porous networks with adjustable pore sizes up to several micrometers by varying the acetonitrile content in aqueous solution used during synthesis. The adjustment of the pore size by variation of the solvent mixture allows for fine‐tuning of the hydrogel and microgel mechanical and diffusion properties with stiffnesses ranging from a few kPa up to more than 100 kPa and diffusion coefficients of 4 kDa FITC‐dextran varying from 50 to over 130 µm^2^ s^−1^. Detailed analyses using optical microscopy, (cryo‐)scanning electron microscopy, rheology, nanoindentation, and fluorescence recovery after photobleaching confirm the dependence of the observed properties on the network morphology and the similarity of the pore size and interconnectivity between hydrogel and microgel samples. The obtained hydrogels and microgels show strong potential for applications in bioengineering, sensing, and responsive materials.

## Graphical abstract

The cononsolvency of poly(dimethyl acrylamide‐*co*‐hydroxyethyl acrylamide) copolymers in water‐acetonitrile mixtures enables the synthesis of hydrogels with finely tuned porosity. Optimized for droplet‐based microfluidics, this approach facilitates the fabrication of porous microgels with tunable mechanical and diffusion properties, offering precise control over physicochemical properties for application in tissue engineering, sensing or catalysis.

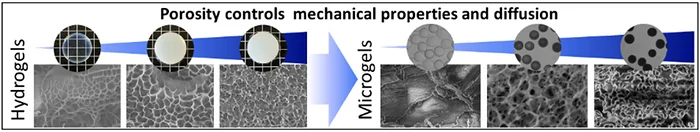

## 1 Introduction

Hydrogels are water‐swollen crosslinked polymer networks that mimic biological tissue like the extracellular matrix, as both contain solid fibers or polymers and a liquid phase, which makes them porous and biphasic [1]. The hydrogel's porous nature enables efficient transport of molecules such as nutrients or metabolites, rendering them interesting for application in biomedicine [2] or catalysis [3]. Porous hydrogels can be classified by their pore size according to the International Union of Pure and Applied Chemistry (IUPAC): Microporous hydrogels are defined as hydrogels with pore sizes below 2 nm, while mesoporous hydrogels exhibit 2 to 50 nm large pores, and hydrogels with pores of more than 50 nm are termed macroporous [4].

Microgels, which are colloidal‐sized hydrogel particles, exhibit an increased surface‐to‐volume ratio and thus, offer even more tunability regarding porosity, permeability, and pore size [5]. This tunability in microgel porosity allows for precise control over mechanical properties [6], molecular diffusion rates [7, 8], and cellular interactions [8]. For example, porous microgels can be assembled into granular hydrogels with interconnected micropores that promote cell migration, proliferation, and improved matrix deposition, which is crucial for tissue engineering and regenerative medicine [9]. Additionally, the adaptable pore size (from nanometers to micrometers) affects many physicochemical properties including particle stiffness [8], elasticity [9], and swelling behavior [10]. Thus, porous hydro‐ and microgels represent a class of materials that enables the customized design of drug delivery systems or catalyst carriers, among others.

A variety of methods for the production of porous hydro‐ and microgels are available that can be categorized into templating and kinetic methods. Among the templating methods, solid‐phase templating, emulsion templating, and foam templating offer pores in the micrometer range even up to several hundred micrometers, while self‐assembled templating facilitates the production of hydrogels with mesopores [11, 12, 13, 14]. For instance, solid porogens can be leached out after polymerization to reveal the porous structure [11, 12], emulsions can be polymerized and the dispersed phase removed [13], and microfluidics can be employed to generate uniform gas bubbles as templates for better control over pore size distribution [14]. Lastly, self‐assembled amphiphilic molecules like surfactants can form nanostructured templates upon polymerization of the continuous phase [11].

The kinetic methods include bubble generation from reaction, gas injection, electrospinning, and freeze‐drying for hydrogels with micrometer‐sized pores, and phase separation, which typically yields pores in the size range of 2–150 nm [11], although larger pore sizes in the micrometer range can be achieved depending on the polymer system, solvent composition, and polymerization conditions [10]. For bubble generation, blowing agents such as water, hydrogen peroxide, or azo‐compounds release CO_2_, O_2_, or N_2_ gas during polymerization to induce porosity [11, 15], while in the gas injection approach, gas is dissolved in the precursor solution and bubbles form in situ during gelation [16]. Electrospinning has been used to produce porous hydrogels from a variety of polymers, and can be combined with gas foaming and freeze‐drying to produce porous composite hydrogels [17, 18]. Moreover, freeze‐drying can be applied to obtain macroporous hydrogels, where the pore size can be tuned by adjusting the freezing rate and temperature [19, 20].

Phase separation can be used for the production of porous hydrogels via changes in temperature or through the addition of a component that induces immiscibility [11]. For instance, *N*‐isopropyl acrylamide (NIPAAm) was polymerized above the LCST of the corresponding PNIPAAm of 31°C to obtain porous hydrogels with a network structure consisting of agglomerated and interconnected microgel particles. During this process, the soluble NIPAAm monomer phase‐separates into PNIPAAm microgels during polymerization [21]. PNIPAAm microgels can also be produced with increased porosity by the addition of salts (e.g., NaCl) to enhance phase separation during the polymerization by the salting out effect of NaCl [22]. Another example for the addition of immiscibility‐inducing components is methacrylated dextran, which exhibits liquid‐liquid immiscibility upon poly(ethylene glycol) addition. The resulting gels are highly porous with bead‐like walls and interconnected pores [23]. Commercially, the nonsolvent‐induced phase separation (NIPS) is also used for the production of porous water treatment membranes [24, 25].

Another way of using NIPS for the synthesis of porous hydrogels is to exploit the cononsolvency of polymers in solvent mixtures. This method offers several advantages, such as precise control over porosity and therefore resulting physicochemical properties by the solvent composition [10], as well as versatility in stimuli‐responsive design, as the material may exhibit responsiveness to solvent composition [26] during and after synthesis. Cononsolvency is a phenomenon where two solvents that individually are good solvents for a polymer result in poor solvent conditions for the polymer when mixed at certain ratios, causing the polymer chains to collapse or precipitate. In contrast, cosolvency is the effect where mixing two poor solvents creates a better solvent. Cononsolvency arises from complex interactions between the solvent, cosolvent, and polymer, including competitive hydrogen bonding [27, 28]. One of the most prominent examples is the sharp coil‐to‐globule transition of poly(*N*‐isopropyl acrylamide) in water‐methanol mixtures [29]. Other polymers that experience cononsolvency include poly(*N*‐vinylcaprolactam) in various water‐alcohol mixtures [28], poly[*N*‐(4‐vinylbenzyl)‐*N*,*N*‐diethylamine] in water‐isopropanol mixtures [30], and poly(*N*,*N*‐dimethylacrylamide) in water‐dioxane mixtures [31]. However, existing cononsolvency‐based porous hydrogel systems are largely concentrated in macroscopic homopolymer networks, where the solvent composition is the primary control parameter for porosity, limiting the tunability of the resulting pore structure and physicochemical properties.

While cononsolvency‐induced phase separation has been explored as a route to porous hydrogels, existing examples are largely concentrated in PNIPAAm‐based homopolymer systems in mixed solvents [10, 32, 33, 34, 35], such as water‐methanol [10], water‐ethanol [34], and water‐DMSO [35]. Classical phase separation routes to porous hydrogels, including thermally induced [36, 37] and solvent‐induced methods [37, 38, 39], are well established, and more recently, controlled polymerization‐induced phase separation has been explored as a route to porous hydrogels with defined morphologies [40, 41]. However, across these approaches, the use of copolymer composition as a programmable and independent design parameter to control cononsolvency and pore formation remains largely unexplored. Herein, we address this knowledge gap by exploiting the cononsolvency of dimethylacrylamide (DMAAm) [42]/hydroxyethylacrylamide (HEAAm) [43] copolymers in water‐acetonitrile mixtures, where both the cosolvent content and the comonomer ratio serve as independent parameters for control over porosity, representing a dual‐parameter design strategy that has not been demonstrated in previous systems. This approach yields highly hydrophilic and biocompatible [42, 43] polymer networks based on two structurally similar but chemically distinct monomers, whose different dimethyl and hydroxyethyl side groups facilitate precise adjustment of the cononsolvency behavior by varying the monomer ratio. We present the synthesis of DMAAm/HEAAm‐based porous hydrogels with tunable porosity and thus adjustable physicochemical properties, and demonstrate the direct translation of this copolymer‐controlled cononsolvency principle to droplet‐based microfluidics for the fabrication of highly porous microgels, where porosity is induced in situ during polymerization within droplets without the need for postprocessing or templating steps. To our knowledge, this is the first report to combine the DMAAm/HEAAm copolymer composition and the cosolvent concentration to program cononsolvency‐induced porosity in a highly hydrophilic and biocompatible hydrogel platform, and to translate that same design concept from bulk hydrogels to droplet‐microfluidic porous microgels within one unified materials framework. The produced materials, with tunable pore architecture, mechanical properties, and diffusion characteristics, hold strong potential for applications in tissue engineering, drug delivery, and the design of stimuli‐responsive materials.

## 2 Results and Discussion

### 2.1 Synthesis and Characterization of Porous Hydrogels

#### 2.1.1 Synthesis, Swelling and Responsiveness

To determine which DMAAm:HEAAm ratio and acetonitrile fraction are suitable for the synthesis of porous hydrogels, cloud point temperature measurements were performed (Figure 1A). For this, linear DMAAm‐based copolymers were synthesized with 0 and 20% HEAAm content. The cloud point temperature, i.e., the temperature at which the solution turns turbid, was determined for both polymers at varying acetonitrile fractions ranging from 65 to 95 vol%. In polymers like PNIPAAm, an LCST‐type phase behavior can be observed, where the LCST is lowered to temperatures of below 0°C for intermediate water‐methanol compositions [44]. In contrast, PDMAAm exhibits a UCST‐type phase behavior where phase separation occurs upon cooling the solution [31]. For PDMAAm homopolymers, the cloud point temperature increases when the acetonitrile fraction is raised above 65 vol%, peaks at roughly 35°C at 85 vol% acetonitrile, and then decreases again at even higher acetonitrile fractions. For the copolymer that contains 20% HEAAm, the maximum cloud point temperature is shifted to approximately 50°C at 90 vol% acetonitrile. Note that the observed differences in the cloud point temperature determined visually or by turbidimetry are likely caused by kinetic effects, as heating is much slower for the visual determination of the cloud point temperature, allowing more time for equilibration and phase separation during cooling. The hydroxyethyl side chain of HEAAm is more polar than the methyl groups in DMAAm, which increases the temperature required for polymer dissolution at high acetonitrile fractions. A similar behavior has been observed for PDMAAm in water‐dioxane mixtures [31].

**FIGURE 1:**
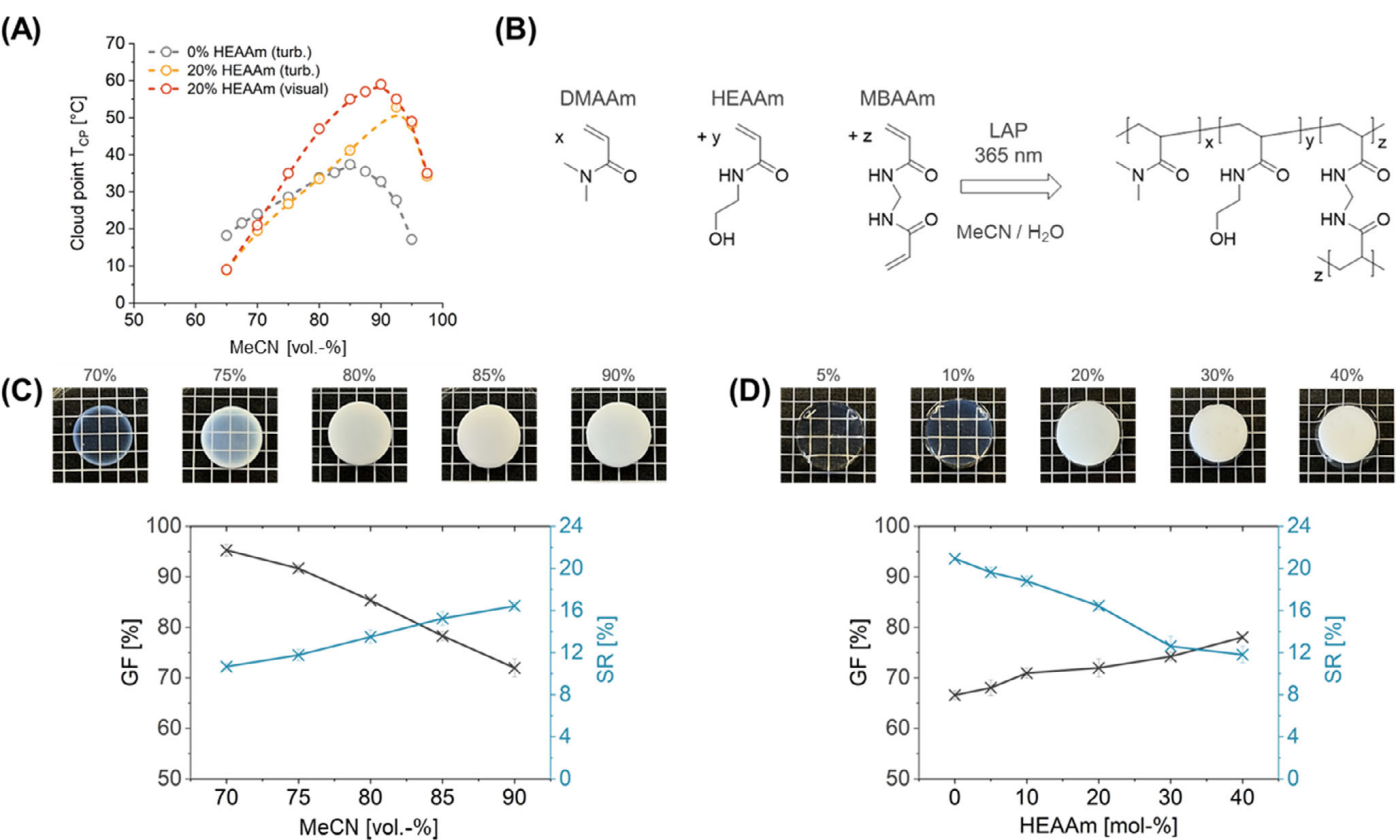
(A) Cloud point temperature (*T*_CP_) measurements of DMAAm/HEAAm mixtures at different ratios of water and acetonitrile (MeCN) (turbidity is determined from the turbidimetry cooling curve and by visual inspection of the solutions). (B) Overview of the reaction for the formation of hydrogels and microgels employing the monomers DMAAm and HEAAm, the crosslinker MBAAm and the photo‐initiator LAP in water‐acetonitrile mixtures. Photographic images, gel fractions (GF) and swelling ratios (SR) of hydrogels synthesized at (C) 20 mol% HEAAm and varying acetonitrile fractions, and (D) 90% acetonitrile varying HEAAm contents.

Based on these cononsolvency results, hydrogels were synthesized with an acetonitrile fraction ranging from 70 to 90 vol% and HEAAm contents around 20 mol% (varied from 0 to 40 mol%). Since the synthesis is performed at room temperature, varying these parameters within the determined ranges should facilitate control over the cononsolvency and the resulting hydrogel porosity, as for certain polymer and solvent compositions the monomers are soluble while the formed polymer is insoluble as it is below its UCST cloud point temperature. In addition to DMAAm and HEAAm, the acrylamide‐based crosslinker *N*,*N*’methylenebisacrylamide (MBAAm) and the photoinitiator lithium phenyl‐2,4,6‐trimethylbenzoylphosphinate (LAP) were employed for the formation of hydrogels (Figure 1B). All reactants were dissolved in the water‐acetonitrile mixtures, and the solutions were homogenized, filled into molds and irradiated with UV light at 365 nm to induce polymerization.

After the synthesis of the hydrogels, photographic images were taken as seen in Figure 1C,D. At a constant HEAAm content of 20 mol%, the hydrogels appear translucent at low acetonitrile fractions and more opaque with an increasing acetonitrile fraction used during the synthesis (Figure 1C). Accordingly, the gel fraction (GF) decreases from approximately 95% at 70 vol% acetonitrile to close to 70% at 90 vol% acetonitrile. Most likely, the polymerization is inhibited at high acetonitrile fractions because the turbidity of the solution or hydrogel leads to scattering of the UV light and thus, inhomogeneous or incomplete irradiation [45]. The swelling ratio (SR), on the other hand, experiences an increase with increasing acetonitrile content. This can most likely be attributed to the concurrent decrease in gel fraction, which reflects a less densely crosslinked network with a higher capacity for water uptake [46, 47].

Next, the acetonitrile fraction was kept at 90 vol%, while the HEAAm content was increased from 0 to 40 mol%. Even though the hydrogels became turbid at higher HEAAm contents, the gel fraction and swelling ratio showed an inverse trend. These observations are likely related the hydrogen‐bond‐donating ability of HEAAm enabling stronger interchain polymer interactions, thereby better retaining the polymer network (higher GF) and leading to a denser network that restricts the swelling (lower SR) [48]. To confirm these hypotheses and further elucidate the relationship between the hydrogel structure and their properties, the hydrogels are characterized using electron microscopy and rheological measurements.

#### 2.1.2 Structural and Mechanical Properties

Scanning electron microscopy (SEM) enables imaging of the network morphology, including pore size and shape, polymer network thickness, and pore interconnectivity. SEM images for five selected samples with varying HEAAm and acetonitrile contents are shown in Figure 2A. While there is a slight fibrous structure on the sample produced at 20% HEAAm and 70 vol% acetonitrile (H20A70), there are no visible pores penetrating into the material. This coincides with the translucent appearance of the hydrogel. In contrast, the samples produced at 80 and 90 vol% acetonitrile (and 20% HEAAm) do exhibit big macropores with sizes in the micrometer range that go deeper into the material. At 80 vol% acetonitrile, the polymer network forms smooth and thin walls in between the pores. However, at high acetonitrile fractions of 90% and with increasing HEAAm content (20 and 40 mol%) the polymer network becomes increasingly heterogeneous and granular. Especially in the sample with the highest HEAAm and acetonitrile concentration (H40/A90), the polymer network consists of small agglomerated interconnected polymer beads. This phenomenon has been described before in dextran methacrylate‐based hydrogels that are crosslinked in the presence of poly(ethylene glycol) to induce phase separation during gelation [23].

**FIGURE 2:**
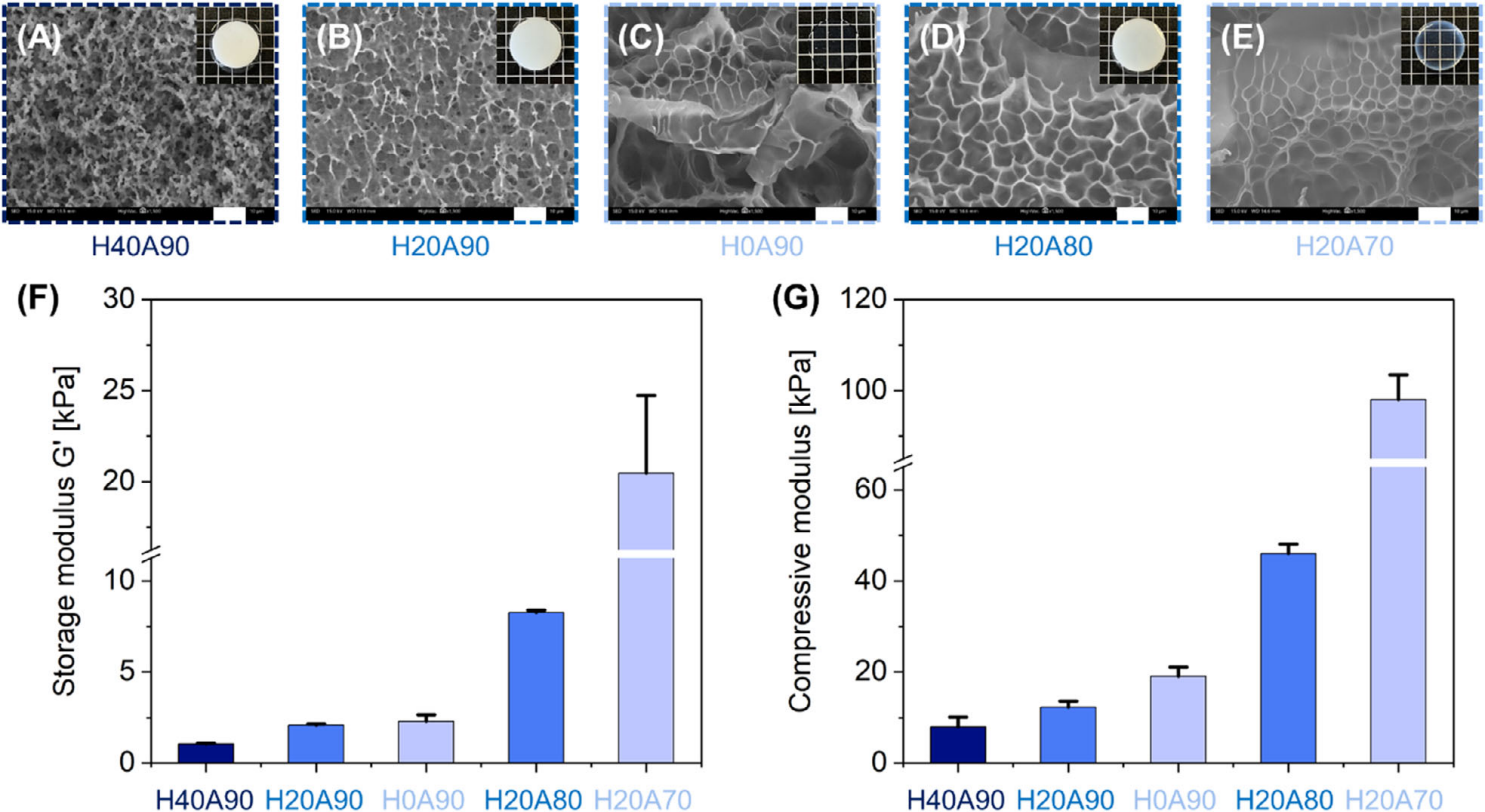
Photographic images and cryo‐SEM images taken of hydrogels produced at varying HEAAm concentrations (A, B, and C) and at varying acetonitrile fractions (B, D, and E) as indicated below the respective images. The abbreviations HXXAYY denote the HEAAm concentration XX (in mol%) and acetonitrile content YY (in vol %). Scale bars represent 10 µm. Graphs show results for the rheological determination of (F) the storage modulus and (G) the compressive modulus of the produced hydrogels (see also Figure S1).

Rheological measurements for the storage modulus and compression tests were performed to determine the influence of the network morphology on the mechanical properties. The storage modulus reflects the elastic response of the hydrogel network [49, 50, 51]. The compressive modulus amounts to approximately three times the storage modulus across all samples [52, 53], consistent with the behavior of an incompressible material under undrained conditions, as the timescale of compression is significantly shorter than the time required for water to fully escape the network. The compressive modulus therefore confirms the storage modulus values.

Hydrogels that were synthesized at 80 and 70 vol% acetonitrile are the stiffest with storage moduli of roughly 7 and 22 kPa, respectively. Their compressive moduli amount to 40 and 100 kPa. As the measurements were performed under drained conditions, the compressive modulus reflects the intrinsic stiffness of the polymer network and is therefore complementary to the nanoindentation data, which probes the mechanical response at the hydrogel surface. At a constant acetonitrile concentration of 90 vol%, the moduli are significantly lower, with values below 3 and 20 kPa, and decrease with increasing HEAAm content or increasing porosity. Similar observations have been made for polyvinyl alcohol/hydroxyapatite/tannic acid hydrogels where a decrease in pore size leads to increased elastic moduli, with the increased mechanical stability primarily attributed to an increased number of hydrogen bonds [47]. In our hydrogel system, where HEAAm content varies between samples (H40A90, H20A90 and H0A90), an analogous contribution of hydrogen bonding to the mechanical properties cannot be excluded and may partly account for the observed trends. However, it should be noted that for the samples where the HEAAm content is identical across all compositions (all H20 samples, as well as microgel samples discussed below), hydrogen bonding contributions can be considered constant and are therefore unlikely to account for the differences in mechanical properties between microgel samples, which are instead primarily governed by pore morphology and crosslink density. Generally, hydrogels with larger pores are softer due to bigger void spaces that deform under pressure [54]. Nonetheless, mechanical properties are influenced by a variety of factors such as hydrogel network composition, pore diameter and crosslinking density [55].

### 2.2 Synthesis and Characterization of Porous Microgels

#### 2.2.1 Synthesis, Swelling and Responsiveness

After the synthesis of porous hydrogels based on DMAAm and HEAAm was established, the method was transferred to a droplet‐based microfluidic setup. Microfluidic production of microgels inherently provides a high degree of reproducibility, as droplet generation is controlled by the flow rates of the continuous and dispersed phases and the channel geometry. Minor batch‐to‐batch variations may arise from small differences in the weighed masses of the individual components; however, the precise control over the droplet generation process ensures consistently narrow size distributions across batches [56].

First experiments were performed with the same solution composition that was used for the hydrogel synthesis. However, these experiments did not yield stable microgels. While increasing the LAP concentration to 0.4 mg mL^−1^ helped obtain stable microgels, a core‐shell structure could be observed as a result of oxygen inhibition [57]. Therefore, the LAP content was increased to approximately three times the amount used for the hydrogel synthesis, or 10 mg mL^−1^, which is a value known to yield stable and homogeneously crosslinked microgels [22, 57, 58]. The amounts of DMAAm, HEAAm and MBAAm as well as the cononsolvent contents remained the same to ensure comparability between the hydrogel and microgel samples.

The droplet‐based microfluidic setup for the optimized synthesis is shown in Figure 3A. Hydrofluoroether (HFE) oil with 2 wt% FluoSurf was used as the continuous phase. FluoSurf is a neutral surfactant that stabilizes the formed water‐in‐oil emulsion until polymerization. The dispersed phase consisted of an aqueous acetonitrile solution with 0, 80, or 90 vol% acetonitrile that contained the aforementioned reactants. On the microfluidic chip, droplets of this solution were sheared off by the continuous phase at the cross junction. The droplets were thoroughly mixed in the serpent‐shaped mixing section and lastly collected at the outlet. The emulsion was irradiated in a glass vial with UV light for 10 s to fully polymerize the monomers inside the droplets and form crosslinked microgels. The irradiation time is reduced compared to the hydrogel synthesis due to the smaller size and total volume of the emulsion droplets, as well as the possibility to shake the emulsion during irradiation. If not stated otherwise, the characterization of the microgels was always performed in aqueous solution after purification.

**FIGURE 3:**
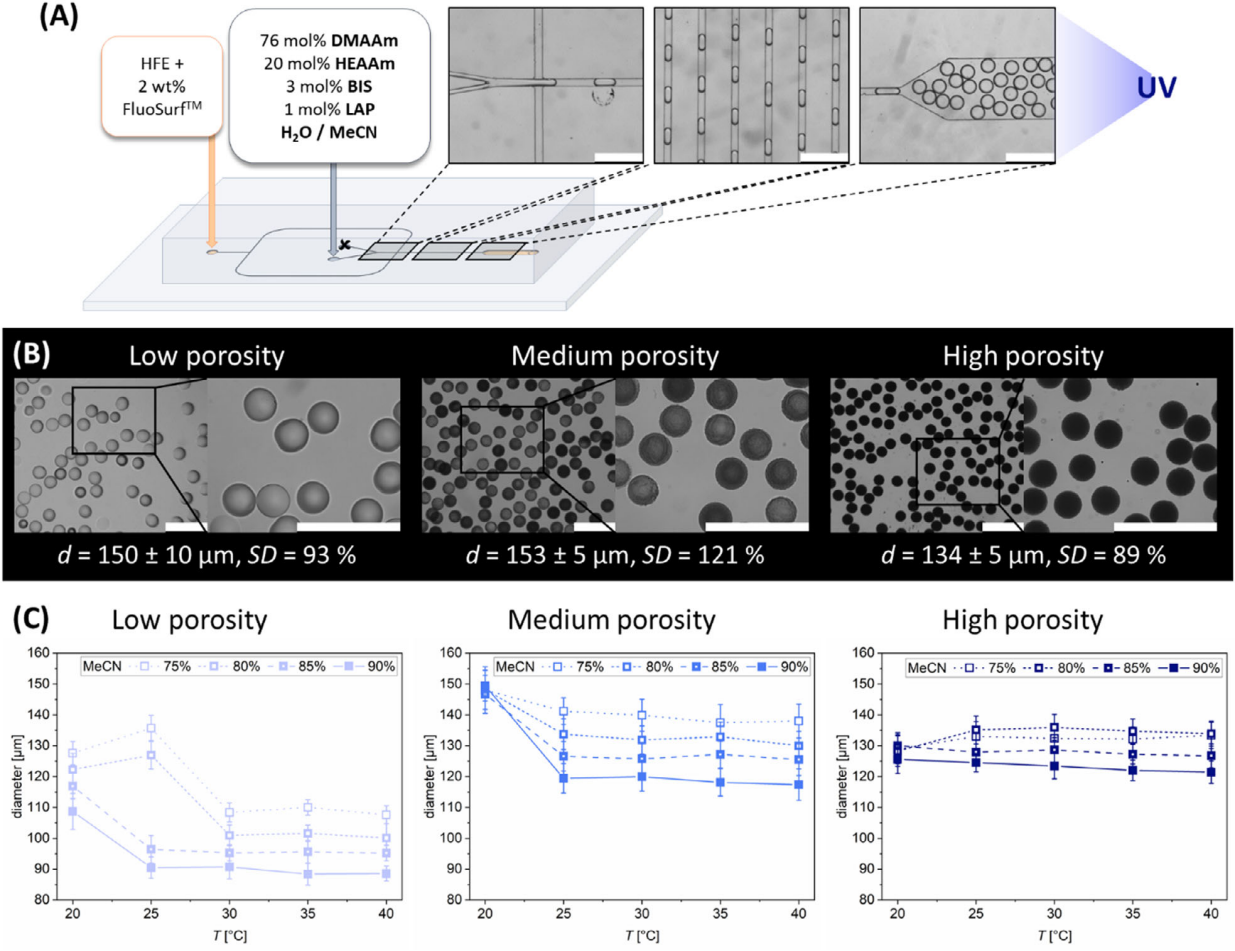
(A) Droplet‐based microfluidic set‐up for the fabrication of porous microgels. Droplets of the aqueous phase containing the monomers, the crosslinker, and the initiator in mixtures of water and acetonitrile (MeCN) are formed in the continuous phase, HFE oil with 2 wt% FluoSurf. The droplets are thoroughly mixed in the serpent‐shaped mixing section before the emulsion is collected at the outlet and irradiated with UV light to initiate the polymerization. (B) Optical microscopy images of the low, medium, and high porosity microgel samples with respective diameters d and swelling degrees SD. Scale bars represent 500 µm, and diameters are given as average ± standard deviation. (C) Sizes of the microgels at different temperatures and acetonitrile concentrations.

With the described droplet‐based microfluidic synthesis, three different samples were produced: a low, medium and high porosity sample fabricated at 0, 80, and 90 vol% acetonitrile content, respectively. As seen in Figure 3B, the microgels turn progressively more opaque and darker with increasing acetonitrile content used during synthesis to enhance the cononsolvency effect, because they become increasingly porous and scatter more light. All three samples exhibit average diameters d of around 130 to 150 µm with very low standard deviations of 10 µm and below confirming a narrow size distribution for all samples (Figure S1). The swelling degrees SD vary between around 90 and 120% and do not follow a trend. However, since the swelling degrees are determined from the diameters in the swollen and in the dried state (Figure S2), it is possible that the microgels with the lowest porosity exhibit lower measured swelling degrees because they may adhere more to the glass slide during drying as a result of their smoother surface and increased contact area (as shown in Section 2.2.2).

The average sizes were additionally broken down into the size distributions that can be found in Figure S1 and Figure S2A. Again, the narrow size distributions show that the samples are highly monodisperse. The microgels with the highest porosity exhibit the smallest diameters. The medium and high porosity samples exhibit significantly bigger pores than the low porosity microgels (as shown in Section 2.2.2). Previously, it has been reported that porous microgels produced through (solid‐phase) templating follow the opposite trend and swell more with increasing pore sizes, leading to bigger microgel diameters. Specifically, Bulut et al. adjusted the mesopore size in their microgels through variation of the prepolymer concentration during synthesis. The swelling degrees were determined under equivalent conditions of purification, swelling in water, and subsequent drying [7]. While in both cases the pores are produced during the synthesis or more precisely during the irradiation and polymerization, herein the pore morphology is governed by the cononsolvency‐induced phase separation rather than monomer or polymer concentration alone. Unlike the variation of prepolymer concentration, which primarily affects the mesh size while maintaining a similar overall structure, the cononsolvency‐induced phase separation in our system fundamentally alters the microgel pore wall structure (as shown in Section 2.2.2). The resulting denser phase‐separated polymer network in the high‐porosity sample reduces the flexibility and thus, inhibits water uptake and yields smaller microgels after purification [10]. All investigated microgels can be redispersed in water after drying and regain nearly 100% of their original size (Figure S2B).

Lastly, the microgels were tested regarding their temperature‐responsive behavior in the presence of acetonitrile (Figure 3C). For this, each sample was dispersed in water‐acetonitrile mixtures at different ratios and then heated to a variety of temperatures. In the low porosity sample, differences in microgel size depending on the acetonitrile content are already visible at 20°C. Interestingly, the microgels in 75 and 80% acetonitrile only collapse significantly at 30°C, whereas those in 85 and 90% acetonitrile already collapse at 25°C. After this initial collapse, the size differences depending on acetonitrile content persist, but no further decrease in size is observed with increasing temperature. In the medium porosity sample, all microgels exhibit a comparable size at 20°C regardless of the acetonitrile fraction. Upon increasing the temperature to 25°C, the microgels collapse from approximately 150 µm to 120 – 140 µm, where again a higher acetonitrile fraction results in smaller microgels. Lastly, in the high porosity sample, only minor differences in microgel size are observed depending on the acetonitrile content, and the temperature‐responsive behavior is no longer present. This indicates that the sample with the highest porosity is less responsive, confirming that these microgels swell less than their less porous counterparts. Even though this observation is the opposite to what the group by Wessling observed for PNIPAAm microgels synthesized in the presence of methanol [10], this difference is likely attributable to the distinct polymer systems and solvent combinations involved: while PNIPAAm exhibits a well‐defined LCST in water‐methanol mixtures, the cononsolvency behavior of our system in water‐acetonitrile may lead to different network responses upon solvent exchange and heating. In the low porosity microgels, the dense and homogeneous network structure limits the extent of swelling and collapse, while the more open and fibrous structure of the high porosity microgels, combined with the larger pores, reduces the overall driving force for thermoresponsive volume changes.

Overall, the porosity of the microgels obtained from droplet‐based microfluidics can be tuned through the variation of the cononsolvent content used during synthesis, which enables control over microgel size, swelling degree, and responsiveness.

#### 2.2.2 Structural and Mechanical Properties

After demonstrating that the use of the cononsolvent acetonitrile during the microfluidic synthesis has an influence on the microgel size, swelling degree and responsive behavior, the samples were analyzed in more detail regarding their structural and mechanical properties.

First, cryo‐SEM was performed for all three samples. For cryo‐SEM, the microgel dispersions were transferred to a sample holder, which was subsequently frozen in liquid nitrogen. The surface was removed to expose a cross‐section of the microgels. The ice on the sample surface and inside the microgel was then sublimated before imaging to visualize the microgels in their swollen state and to give insight on their internal structure. The cryo‐SEM images at 600 × and 8000 × magnification are shown in Figure 4A. Additional images at 2000 × and 4000 x magnification can be found in Figure S3. For the lowest porosity, the microgels exhibit a smooth network structure with some fractures visible from breaking off the top of the microgel. The network structure is less porous than what has been observed for the hydrogels because of the complete absence of acetonitrile during microgel production. In the medium porosity sample, very big pores in the micrometer range (1.46 ± 0.30 µm) are visible. Even though the microgel is mostly homogeneous in structure, it does have a looser crosslinked shell that contains some bigger pores. Additionally, separate polymer walls with thicknesses of 0.24 ± 0.07 µm can be seen in the images. These polymer walls are even thicker in the high porosity sample with 0.73 ± 0.09 µm in which the walls seem to consist of polymer agglomerates that coagulate during the polymerization as a result of the high cononsolvent content. This also leads to an elevated pore size of 1.88 ± 0.52 µm. The pore size and pore wall thickness distributions are shown in more detail in Figure S4.

**FIGURE 4:**
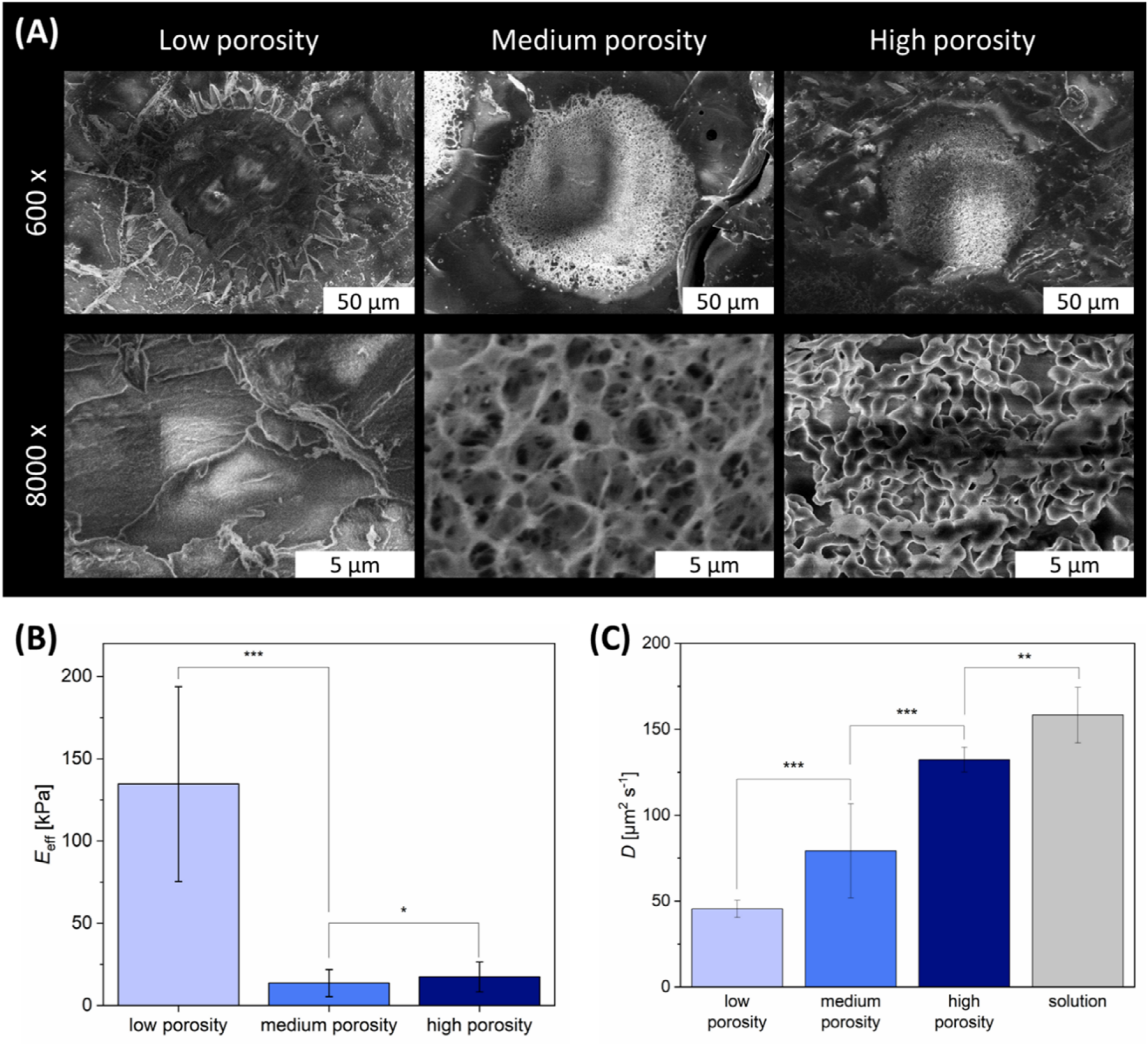
(A) Cryo‐SEM images of the low, medium and high porosity microgels at 600× and 8000× magnification. (B) Effective Young's moduli of the microgels measured via nanoindentation (*n* ≥ 50). (C) Diffusion coefficients of 4 kDa FITC‐dextran measured via FRAP in CLSM (*n* = 10 for microgels, *n* = 5 for solution). The statistical significance is denoted as ^*^(*p* < 0.05), ^**^(*p *< 0.01), ^***^(*p* < 0.001), N.S. (= no significance, *p* > 0.05).

For the investigation of the mechanical properties, nanoindentation measurements were performed (Figure 4B). With this technique, microgels are indented with a probe to determine the effective Young's modulus $E_{\mathrm{eff}}$ from the deformation. It is important to note that the microgels are only indented around 1 to 2 µm, so the technique mainly gives insight into the properties of the microgel surface. Since a more loosely crosslinked corona can be observed for the more porous microgels, it can be assumed that the mechanical properties are slightly different deeper inside the microgels and therefore also depend on the analytical technique used (i.e., rheology or nanoindentation). The statistical analysis was performed using Welch's t‐test. The low porosity microgels are very stiff with an average effective Young's modulus of roughly 135 ± 60 kPa, and thus, approximately 8 – 10 times stiffer than the other two samples, a difference that is highly statistically significant (*p* < 0.001). The medium and high porosity samples are considerably softer, with values of approximately 14 ± 8 and 17 ± 9 kPa, respectively. Although a statistically significant difference between the two was detected (*p* = 0.019), the large standard deviations and the small difference in average values suggest that the mechanical properties of the two samples are in a comparable range, likely reflecting the inherent variability of nanoindentation measurements on soft, fibrous materials with fuzzy coronas as observed in cryo‐SEM. The significant difference in stiffness between the low and medium porosity samples, as well as the comparable values of the medium and high porosity samples, can be explained by the acetonitrile fractions used during synthesis: the low porosity sample was produced without any acetonitrile, whereas the medium and high porosity samples were produced with similar acetonitrile fractions of 80 and 90%, respectively. Consistently, nanoindentation results obtained for the hydrogels confirm that an intermediate acetonitrile fraction of 70% yields an intermediate stiffness of around 43 ± 8 kPa (Figure S5). The more fibrous structure of the medium and high porosity microgels seen in cryo‐SEM offers space for possible deformations to occur, leading to a more flexible and softer network. In contrast, the highly homogeneous crosslinking throughout the low porosity microgel results in a significantly stiffer material. It is worth noting that since the HEAAm content is identical across all microgel samples, hydrogen bonding contributions can be considered constant and are therefore unlikely to account for the differences in mechanical properties observed between the samples. Instead, the stiffness differences are primarily attributed to the pore morphology and network structure resulting from the acetonitrile fraction used during synthesis, which is further supported by the gel fraction data obtained for the corresponding hydrogel compositions (Figure 1), decreasing from 95% for the lowest porosity (produced at 70% acetonitrile) to 70% for the high porosity sample (produced at 90% acetonitrile), indicating a lower crosslink density in the more porous samples.

Lastly, while permeability assays remain inconclusive (Figure S6), fluorescence recovery after photobleaching (FRAP) experiments (Figure 4C) were performed to determine the diffusion coefficients of a 4 kDa FITC‐labelled dextran solution inside the three microgel samples (as well as the pure solution as reference) (Figure S7). The statistical analysis was performed using the Mann‐Whitney test. The diffusion coefficient increases with increasing porosity from 46 ± 5 µm^2^ s^−1^ for the lowest porosity to 79 ± 27 µm^2^ s^−1^ for the medium porosity sample and to 132 ± 7 µm^2^ s^−1^ for the high porosity sample. The differences between all groups are highly statistically significant (*p* < 0.001 for low vs. medium and medium vs. high porosity microgels). The pure FITC‐dextran solution has a diffusion coefficient of roughly 160 ± 16 µm^2^ s^−1^, which is approximately 20% higher than that of the high porosity sample, and the difference remains statistically significant (*p* = 0.004), confirming that even at the highest porosity, diffusion inside the microgels is slightly more restricted than in free FITC‐dextran solution. Therefore, these results demonstrate that increasing porosity facilitates diffusion inside the microgels, with diffusion coefficients that can be tuned and approach values close to that of the free solution.

These results of the structural and mechanical characterization coincide with the average diameters and swelling degrees observed before: with increasing cononsolvent content, the polymer walls become thicker, which leads to an increase in pore sizes, and therefore an increase in diffusion coefficient and a decrease in stiffness.

### 2.3 Comparison of Porous Hydrogels and Microgels

The porous hydrogels and microgels produced using the presented approach have been imaged using scanning electron microscopy and characterized regarding their mechanical properties to enable direct comparison of both hydrogel materials at both length scales (Figure 5). Additionally, the average pore sizes and pore wall thicknesses for the hydro‐ and microgels were determined from SEM images and displayed in Table 1 along with the respective standard deviations.

**FIGURE 5:**
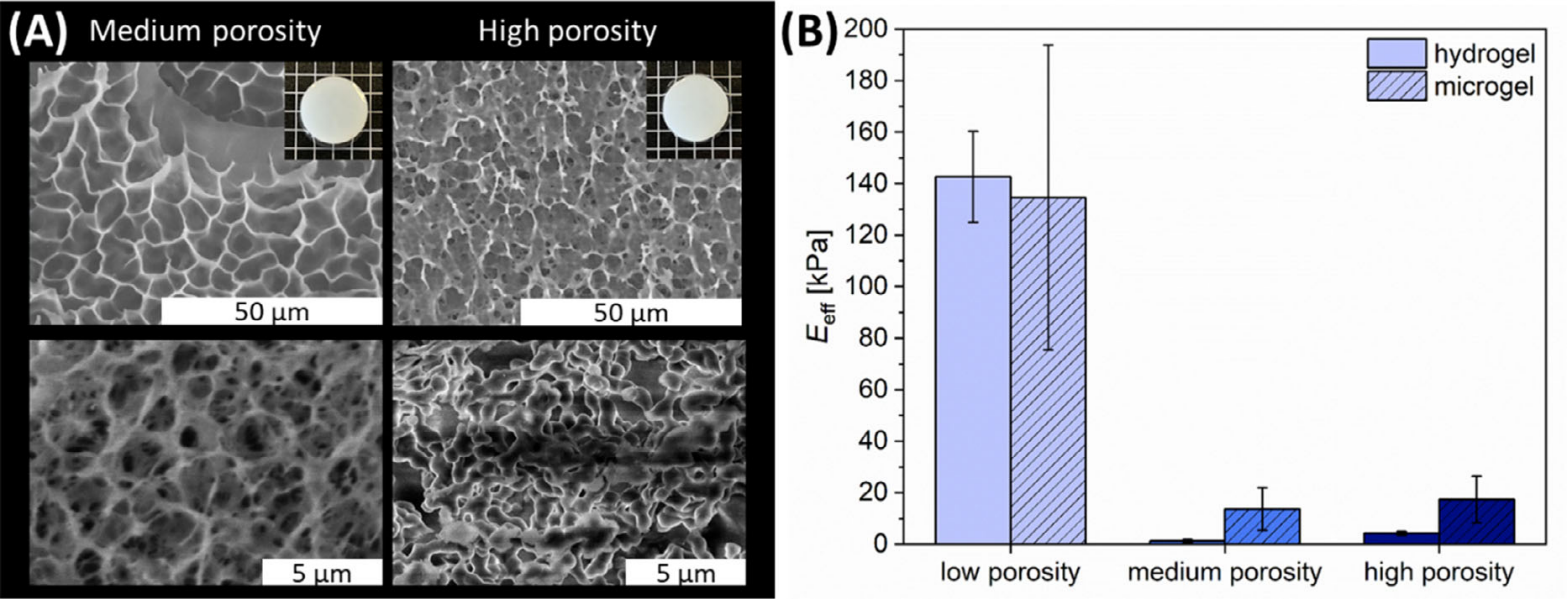
(A) Cryo‐SEM images for the hydrogels (top) and microgels (bottom) at medium and high porosity. (B) Nanoindentation results for both the hydro‐ and microgels for direct comparison.

**TABLE 1:** Average pore size and pore wall thickness for the hydro‐ and microgel samples at three different porosities with respective standard deviation.

	hydrogels		microgels	
porosity	pores	walls	pores	walls
low	^a^	0.31 ± 0.09 µm	^a^	^a^
medium	4.38 ± 1.33 µm	0.45 ± 0.14 µm	1.46 ± 0.30 µm	0.24 ± 0.07 µm
high	3.52 ± 2.08 µm	0.61 ± 0.28 µm	1.88 ± 0.52 µm	0.73 ± 0.09 µm

^a^ No values could be determined because of the absence of measurable pores or pore walls.

Not only do both the hydrogels and microgels appear increasingly turbid with increasing acetonitrile fractions, but the network structure is very similar as well. While the medium porosity samples with 20 mol% HEAAm and 80 vol% acetonitrile form a smooth and fibrous structure, the high porosity gels with 20 mol% HEAAm and 90 vol% consist of agglomerated globular polymer walls.

Nonetheless, the overall pore size in the hydrogels is roughly two to three times larger than that of the microgels (Table 1). One contributing factor may be the more homogeneous irradiation and thus more uniform polymerization in the microgels due to their smaller size. Additionally, the higher photoinitiator concentration used for the microgel synthesis influences the kinetics of the polymerization: An increased LAP concentration leads to a higher number of initiation sites, which is expected to result in shorter polymer chain lengths and may alter the kinetics of the cononsolvency‐induced phase separation during the polymerization, ultimately leading to differences in network structure between hydrogels and microgels. For the microgels, no pore size could be determined for the low porosity sample, while the medium and high porosity microgels exhibit pore sizes in the micrometer range of 1.46 and 1.88 µm, respectively. The high porosity hydrogel exhibits greater heterogeneity in pore size and shape compared to its microgel counterpart, as reflected by the larger standard deviation in pore size (2.08 vs. 0.52 µm), with a broader distribution ranging from smaller pores comparable to those in the medium porosity microgel to pores exceeding those observed in the medium porosity hydrogel.

The pore wall thicknesses are similar in the hydrogels and microgels. The pore wall thickness varies between approximately 0.25 µm and 0.75 µm and continuously increases with increasing porosity because of the phase separation and agglomeration of the polymer during polymerization. The standard deviation for the pore wall thickness increases accordingly, reflecting the greater heterogeneity of the pore walls at higher porosities. The standard deviations for both the pore size and the pore wall thickness are smaller in the microgels (pore size: 0.30 and 0.52 µm; pore wall thickness: 0.07 and 0.09 µm) compared to the hydrogels (pore size: 1.33 and 2.08 µm; pore wall thickness: 0.09–0.28 µm), most likely because of the increased control and sample homogeneity achieved during microfluidic synthesis. This suggests that while the higher LAP concentration used for microgel synthesis influences polymerization kinetics and network structure as discussed above, it simultaneously promotes a more uniform network formation in combination with the more controlled synthesis approach, as evidenced by the narrower distributions of both pore size and pore wall thickness.

To ensure comparability of data for the mechanical properties, both hydro‐ and microgels were measured with nanoindentation. The effective Young's modulus $E_{\mathrm{eff}}$ that is determined this way differs from rheological measurement data as a result of the difference in techniques: in rheology the whole gel is deformed, while in nanoindentation only about 1 µm of the surface is indented. Nonetheless, the compression tests (Figure 2G) do show values that are comparable to the effective Young's modulus determined via nanoindentation, suggesting that the surface mechanical properties probed by nanoindentation are representative of the bulk network properties, and that the mechanical response of these hydrogels is largely governed by the polymer network structure rather than surface‐specific effects.

Especially in the low‐porosity samples without macropores in the micrometer range, the network structure is much more homogeneous and differs a lot from the medium‐ and high‐porosity samples. Therefore, the effective Young's modulus of the low porosity samples significantly differs from the other two. Even though the values measured for the hydrogels differ slightly from the values measured for the microgels, the overall trend is the same. This confirms that the cononsolvency of DMAAm/HEAAm polymers in water‐acetonitrile mixtures can be utilized for the production of hydrogels across a wide range of length scales from micrometers to centimeters.

## 3 Conclusions

In the present work, we investigated the cononsolvency of dimethyl acrylamide (DMAAm)‐based polymers and dimethyl acrylamide/hydroxyl ethyl acrylamide (HEAAm) copolymers in water‐acetonitrile mixtures. We found that with increasing HEAAm content and high acetonitrile fractions the polymers undergo phase transition even at room temperature, which can be utilized for the fabrication of porous hydrogels as the monomers are soluble in the solvent mixtures while the formed polymers collapse and phase separate to form interconnected agglomerates.

While the hydrogels got progressively more opaque with increasing HEAAm and acetonitrile contents as a result of the porous structure and associated scattering of light, their stiffness decreased. Scanning electron microscopy revealed that the porous hydrogels contained macropores in the micrometer range and granular pore walls.

After optimization, the synthesis could be translated to droplet‐based microfluidics, which was used for the fabrication of microgels with controlled porosity. The more porous microgels exhibit a darker appearance and more fuzzy surface as seen in optical microscopy. The rough and fuzzy surface is also visible in cryo‐scanning electron microscopy images, which confirmed homogeneous crosslinking for the low‐porosity sample and the presence of micrometer‐sized pores for the medium and high‐porosity microgels. Especially in the high porosity microgel, the agglomerated polymer strands and beads that make up the microgel network are visible. The temperature‐responsive behavior that could be observed for the low porosity microgels in aqueous solutions with high acetonitrile fractions is diminished in the high porosity microgels, possibly due to the thick polymer pore walls that inhibit the expansion and contraction of pores. Nanoindentation measurements showed that with increasing pore size the microgels become softer (like the hydrogels), and fluorescence recovery after photobleaching experiments revealed that the diffusion coefficient of 4 kDa FITC‐dextran increases with increasing pore size.

Overall, the cononsolvency‐assisted fabrication of porous DMAAm/HEAAm‐based hydro‐ and microgels can be used across different length scales from micrometers to centimeters and offers control over porosity, diffusion, and mechanical properties. With this method, hydrogel materials of different sizes can be tailored for specific applications including catalysis, sensing, or biomedical applications like tissue engineering.

## 4 Experimental Section/Methods

### 4.1 Materials

*N*, *N*’‐methylenebisacrylamide (MBAAm, 99%), *N*,*N*‐dimethylacrylamide (99%, contains 500 ppm monomethyl ether hydroquinone as inhibitor), hydroxyethyl acrylamide (HEAAm, 97%), lithium phenyl‐2,4,6‐trimethylbenzoylphosphinate (LAP, ≥95%) and fluorescein isothiocyanate–dextrans (FITC‐dextrans) were purchased from Sigma‐Aldrich (Merck). Water (HPLC‐grade, CHEMSOLUTE) and acetonitrile (≥99.5%) were purchased from Th. Geyer and Fisher Chemicals, respectively.

### 4.2 Synthesis of Hydrogels

For the hydrogel synthesis, MBAAm (14.5 mg, 0.094 mmol, 3 mol%), DMAAm and HEAAm were dissolved in acetonitrile before adding an aqueous LAP (2.7 mg, 0.01 mmol, 0.3 mol%) stock solution. The amount of acetonitrile and water used was chosen according to the desired water‐acetonitrile composition and to amount to 1 mL volume in total. The amounts of DMAAm and HEAAm used for the different samples can be found in Table 2.

**TABLE 2:** Volumes, masses, amounts and molar fractions of DMAAm and HEAAm used for the synthesis of hydrogels with varying HEAAm contents.

%(HEAAm)	DMAAm				HEAAm			
	*V* [µL]	*m* [mg]	*n* [mmol]	*x* [mol%]	*V* [µL]	*m* [mg]	*n* [mmol]	*x* [mol%]
0	310	296	3.0	100	0	0	0	0
20	248	237	2.4	80	62.5	69.4	0.6	20
40	186	178	1.8	60	125.0	138.8	1.2	40

The 300 µL of this precursor solution is transferred into the circular cavity of the lid of a snap cap vial (12 mm diameter) and immediately irradiated with 365 nm UV light for 30 s at 4 cm distance. The hydrogels were placed in deionized water, which was replaced several times over at least three days to remove unreacted chemicals.

### 4.3 Gel Fraction

For the determination of the gel fraction, the hydrogels were weighed after purification and drying, and the obtained mass was divided by the total mass of the components used for their synthesis, giving the gel fraction as a mass ratio.

### 4.4 Swelling Ratio

After purification, the swelling ratio was determined as the ratio of the hydrogel diameter in the swollen state to the dried state.

### 4.5 Rheology Measurements

Rheology was measured on an Anton Paar MCR 302 rheometer at room temperature. Measurements were carried out with a gap of 1.5–2.3 mm depending on the thickness of the hydrogel. Amplitude sweeps were conducted at 1 Hz at different gaps until a linear viscoelastic regime was observed between strains of γ  =  0.1–1%. For the determination of the storage moduli, frequency sweeps at strains of 0.1 and 1% and an angular frequency regime ranging from ω  =  0.5 – 50 rad s^−1^ (0.1 – 10 Hz) were conducted. The hydrogels were measured after purification and swelling in excess water.

### 4.6 Compressive Mechanical Properties

Compression tests of the hydrogels were performed using a Tinius‐Olsen H10KT mechanical tester at room temperature. Each sample composition was tested in triplicate to ensure reproducibility. After purification and swelling in excess water, the hydrogels were cut into circular specimens with a diameter of 6 mm. To determine the compressive strength, a compression rate of 5 mm min^−1^ was applied. The compressive modulus was determined from the slope of a straight line fitted to the initial linear portion of the stress‐strain curves, within a strain range of 0%–10%. The applied compression rate of 5 mm min^−^^1^ allows water to freely redistribute within and escape from the hydrogel during compression, such that the determined compressive modulus represents the drained modulus of the polymer network.

### 4.7 Scanning Electron Microscopy (SEM)

The morphology of the hydrogels was analyzed by scanning electron microscopy using a Jeol JCM 7000 scanning electron microscope at an accelerating voltage of 15 kV. For this, the freeze‐dried samples were attached to a metallic sample holder using double‐sided carbon tape. Next, the sample holder was transferred into a gold sputter coater (automatic Au Sputter Coater K550X from Emitech connected with an RV3 two‐stage rotary vane pump), followed by coating for 60 s at 15 mA under vacuum. The resulting samples were analyzed by SEM.

### 4.8 Droplet‐Based Microfluidics Synthesis of Microgels

First, a master mold for the microfluidic device was designed with the AutoCAD software (Autodesk, USA) and printed on a dark‐field photomask at 25 000 dpi. The pattern was then transferred onto an SU‐8 epoxy‐based photoresist, which was attached to a silicon wafer via soft photolithography to obtain the master mold [59]. For the fabrication of the microfluidic device from the master mold, poly(dimethylsiloxane) (PDMS) was mixed with a curing agent (Silgard 184 elastomer kit, Dow Corning USA) at a ratio of 10:1 (PDMS: crosslinker). After degassing, the mixture was transferred to the master mold, which was cured at 60°C for 24 h. The cured PDMS chip was carefully cut from the master mold. Two inlets, one for the oil phase and one for the water phase, as well as one outlet were punched through the microfluidic chip with a biopsy puncher (MicrotoNano, Haarlem, Netherlands, diameter: 0.75 mm). The chip and a glass slide were washed with water and isopropanol three times each, then dried in an oven and bonded together through oxygen plasma bonding (30 mL min^−1^, 100 W, 40 s). Lastly, the channels were filled with Aquapel (Pittsburgh Glass Works USA) to functionalize the channels with a hydrophobic coating. Excess Aquapel was removed using a syringe filled with air. The channel diameter was 80 µm.

For the production of porous microgels, MBAAm (14.5 mg, 0.094 mmol, 3 mol%) and LAP (9 mg, 0.031 mmol, 1 mol%) were dissolved in HPLC‐grade water. Then, DMAAm (238 mg, 248 µL, 2.401 mmol, 76 mol%), HEAAm (72 mg, 65 µL, 0.625 mmol, 20 mol%), and acetonitrile were added. For the low porosity sample, only 1000 µL water was used. For the medium porosity, 80% acetonitrile or 800 µL acetonitrile and 200 µL water were employed. For the sample with the highest porosity, 90% acetonitrile or 900 µL acetonitrile and 100 µL water were mixed. This aqueous mixture, as well as an oil phase consisting of fluorocarbon oil (HFE‐7500) with 2 wt.% FluoSurf surfactant, was transferred to two separate Hamilton syringes (1000 series, GASTIGHT, with luer tip), which were mounted on syringe pumps (PHD Ultra, Harvard Apparatus, Holliston, USA). The syringes were connected to the microfluidic device via fine‐bore polyethylene tubing (inner diameter: 0.40 mm, outer diameter: 1.10 mm). The flow rates were adjusted to 300 µL h^−1^ for the aqueous phase and to 600 µL h^−1^ for the oil phase. Droplet formation could be observed with an inverted microscope (Motic AE2000, TED PELLA Inc., Redding CA) and recorded with an attached camera (Flea3, Point Grey, Richmond CA). The resulting water‐in‐oil emulsion was irradiated with UV light for 10 s (3.6 W, 365 nm). For the purification of the microgels, the solvent was exchanged three times against HFE‐7500 oil, once against n‐heptane, and at least three times against HPLC‐grade water. In between purification steps with water, samples were centrifuged at 8000 rpm for 15 s to accelerate the sedimentation of the microgels.

### 4.9 Brightfield Microscopy

Brightfield microscopy images were taken with an inverted microscope (Olympus CKX53) equipped with a fast camera (Olympus DP23). To determine the size distributions of the microgels, the diameters of at least 50 microgels were measured in ImageJ and plotted in Origin. For the investigation of the responsive behavior, the concentrated microgel dispersions were dispersed in the respective water‐acetonitrile mixture and then heated to the desired temperature with a heating stage (Tokai‐Hit ThermoPlate). At least 50 microgel diameters were measured with ImageJ and the results plotted in Origin.

Swelling degrees SD were determined from the diameters of the microgels in the swollen state $d_{s}$ and dry state $d_{d}$ using Equation (1).

(1) $$SD=\frac{d_{s}-d_{d}}{d_{d}}$$

### 4.10 Cryo‐SEM

For cryo‐SEM, the concentrated microgel samples were transferred to a sample holder, frozen rapidly in liquid nitrogen, and the surface scraped off to make the cross sections accessible for microscopy. The samples were transferred to the preparation chamber under vacuum, where they were scraped off again with a scalpel. The images were taken on a cold field emission SEM (FE‐SEM/S4800) equipped with a Gatan cryo‐Chamber. The samples were measured at 2 µA with 1.0 kV using an SE detector.

### 4.11 Nanoindentation

Nanoindentation measurements were performed on a high‐throughput mechanical screening Nanoindenter Pavone (Optics11Life, Amsterdam, The Netherlands). Each measurement included a minimum of 50 indentations: Hydrogel samples were indented at different locations across their surface and for microgels, separate microgels were indented at their center. Microgel and hydrogel samples were fixed in a well plate with a poly‐L‐Lysine coating. All measurements were performed using a cantilever‐based probe with a spherical tip (10 µm radius, 0.38 N/m stiffness for microgel samples and 49.5 µm radius, 0.41 N/m stiffness for hydrogel samples) in peak load poking mode, aiming for a maximum indentation depth of 16% of the tip radii, respectively. The Hertzian model is used to determine the effective Young's modulus $E_{\mathrm{eff}}$ by fitting the load‐indentation curves. All measurements were conducted in MilliQ water at room temperature on hydrogels and microgels after purification and swelling in excess water.

### 4.12 Confocal Laser Scanning Microscopy (CLSM)/Permeability Assay/Fluorescence Recovery After Photobleaching (FRAP)

The confocal laser scanning microscope used was a Leica TCS SP8 confocal microscope (Leica, Wetzlar, Germany). FITC‐dextrans were excited with an Argon laser at a wavelength of 488 nm (10% laser power) and their fluorescence detected between 500 and 550 nm with a PMT detector. For brightfield images, PMT trans detector was used. For the permeability assay, concentrated microgel dispersions were mixed with 1 mg mL^−1^ FITC‐dextran solutions of varying molecular weights (4, 40, and 250 kDa) at a 1:10 ratio (microgel dispersion:FITC‐dextran solution). After the images were taken, a region of interest was placed through the middle of the microgel in the LAS X software to generate the intensity profiles, which were normalized and plotted in Origin.

For FRAP measurements, 10 microgels were bleached per sample, and 5 spots were bleached for the pure FITC‐dextran solution. For this purpose, a spherical region of interest (ROI) with a diameter of 40 µm was bleached with the argon laser at 488 nm and 100% laser power. 20 images were taken pre‐bleach at 96 ms intervals, 20 images were taken during bleach at 96 ms intervals, and after bleaching 80 images were taken at 96 ms intervals and 30 images were taken at 1 s intervals. In LAS X, three ROIs were used to determine the change in fluorescence in three regions: the bleach point, the complete image and the background (the area in which no bleaching occurred). These were used to perform a background correction and normalize the fluorescence intensity in Origin. The normalized fluorescence recovery curves were plotted and fitted with the Soumpasis fit, which was extended by the term $F_{0}$ because in this case the fluorescence at t  =  0 was not set to 0. The equation used for the Soumpasis fit [60] of the recovery function F(t) is shown in Equation (2) with the fraction of mobile species k, the time t, the characteristic diffusion time $\tau_{D}$ and the zero‐ and first order modified Bessel functions $I_{0}$ and $I_{1}$, respectively.

(2) $$F\left(t\right)=k\cdot e^{\frac{\tau_{D}}{2t}}\left[I_{0}\left(\frac{\tau_{D}}{2t}\right)+I_{1}\left(\frac{\tau_{D}}{2t}\right)\right]+F_{0}$$

The diffusion coefficient D can be calculated from Equation (3) after the radius of the bleach point w is determined from the full width at half maximum from the intensity profile in LAS X.

(3) $$D=\frac{w^{2}}{\tau_{D}}$$

## Author Contributions

**Maria I. Pieper**: conceptualization, investigation, Writing – original draft, methodology, validation, visualization, Writing – review and editing, data curation. **Daniel D. Stöbener**: conceptualization, investigation, writing – review and editing, methodology, validation, visualization, data curation, writing – original draft. **Bernhard Häßel**: investigation, methodology, data curation, validation, writing – review and editing, writing – original draft. **Richard Hoogenboom**: conceptualization, supervision, resources, writing – review and editing. **Andrij Pich**: conceptualization, supervision, resources, writing – review and editing.

## Conflicts of Interest

The authors declare no conflicts of interest.

## Supporting information




**Supporting File**: marc70423‐sup‐0001‐SuppMat.docx.

## Data Availability

The data that support the findings of this study are available from the authors upon reasonable request.
