# Supplementary material for: Cononsolvency‐Assisted Fabrication of Hydrogels and Microgels With Tunable Porosity, Diffusivity and Stiffness
Source: Macromol Rapid Commun. 2026 Sep 5;47(21):e70423. doi: 10.1002/marc.70423 (PMC13741384; doi:10.1002/marc.70423)
Supplement: Supplementary file 1 [file MARC-47-e70423-s001.docx]

Supporting Information

Cononsolvency-Assisted Fabrication of Hydrogels and Microgels with Tunable Porosity, Diffusivity and Stiffness

Maria I. Pieper, Daniel D. Stöbener, Richard Hoogenboom*, Andrij Pich*

For the determination of the storage modulus and the compressive modulus (**Figure 2**), a frequency sweep and a compression test were performed. The results are shown in **Figure S1**.

**Figure S1.** Photographic images and cryo-SEM images taken of hydrogels produced at varying HEAAm concentrations ((A), (B) and (C)) and at varying acetonitrile fractions ((B), (D) and (E)) as indicated below the respective images. The abbreviations HXXAYY denote the HEAAm concentration XX (in mol%) and acetonitrile content YY (in vol %). Scale bars represent 10 µm. Graphs show (F) the frequency sweep and (G) the compressive modulus of the produced hydrogels.

For the determination of swelling degrees and the investigation of the microgels’ ability to reswell, each sample was images in the swollen, dried and rehydrated state. For each sample at each condition, a minimum of 50 microgel diameters were measured. The respective size distributions are shown in Figure S1 below.

**Figure S2.** Size distributions of the low porosity (left column), medium porosity (middle column) and high porosity (right column) microgels in the swollen (top row), dried (middle row) and rehydrated state (bottom row).

For all samples, the microgels exhibit narrow size distributions. Additionally, the microgels decrease significantly in size upon drying and return to their original sizes and size distribution after rehydration.

For better comparison, the size distributions of all three samples in the hydrated state are plotted in one graph (**Figure S2** **(A)**) and the average diameters of the microgels samples in the dispersed, dried and redispersed state are compared in **Figure S2** **(B)**.

**Figure S3.** (A) Size distributions of the low, medium and high porosity sample in the hydrated state and (B) average sizes and standard deviations of the low, medium and high porosity microgel sample in the hydrated, dried and rehydrated state each.

Like the average sizes (**Figure 1**), the size distributions show smaller diameters for the more porous samples. All samples decrease to roughly 50 % of their initial size when drying and return to their initial sizes after rehydration.

In addition to the cryo-SEM images at 600 x and 8000 x magnification in **Figure 4 (A)**, images were also taken at 2000 x and 4000 x magnification (**Figure S3**).

**Figure S4.** Cryo-SEM images at 2000 x and 4000 x magnification of the low, medium and high porosity microgels.

The low porosity sample present with a homogenous and smooth interior, while the medium and high porosity samples exhibit much bigger pores and separate fibers. The fiber thickness and the pore size were determined and plotted as size distributions in **Figure S4**.

**Figure S5.** Distribution of the fiber thickness (left) and the pore size (right) in the medium (light blue) and high porosity microgel (dark blue).

The broad distributions of the fiber thickness and the pore sizes reflect the variations in fiber thickness and pore size that can be seen in the cryo-SEM images (**Figure 4 (A)**, **Figure S3**). While the pore size only increases slightly from the medium to the high porosity sample, the fiber thickness changes drastically: the fiber thickness approximately triples when increases the porosity. The increased fiber thickness also leads to the darker appearance of the more porous microgels in optical microscopy.

In addition to rheology, the hydrogel samples produced with 20 % HEAAm content at varying acetonitrile fractions were measured in nanoindentation to be directly compared to the microgels. The results can be found in **Figure S5**.

**Figure S6.** Nanoindentation results for the hydrogels produced with 20 % HEAmm at varying acetonitrile fractions. H20 indicating 20 % of HEAAm used and AXX describing the fraction XX % of acetonitrile used during the synthesis.

The nanoindentation results for the hydrogels show the same trend that was found in rheology measurements of hydrogels and nanoindentation measurements of microgels. With an increasing amount of acetonitrile, the hydrogels are more porous and become softer.

A permeability assay was performed by mixing the three different microgel samples with FITC-labelled dextran solution of varying molecular weights (4, 40 and 250 kDa). Fluorescence images were taken and the intensity profiles were plotted (**Figure S6**). Unexpectedly, in all samples except for the low porosity sample at 4 kDa FITC-dextran the fluorescence intensity is very low.

**Figure S7.** Permeability assay of the porous microgel sample with 4, 40 and 150 kDa FITC-dextrans and the corresponding intensity profiles.

Nonetheless, the fluorescence intensity in the core of the microgel is high enough for fluorescence recovery after photobleaching (FRAP) experiments to be performed.

The recovery curves for the three investigated microgels as well as the pure 4 kDa FITC-dextran solution are shown in **Figure S7**.

**Figure S8.** Recovery curves of FRAP measurements of 4 kDa FITC-dextrans for the low, medium and high porosity microgels as well as the solution itself.

Even though the fluorescence could only be bleached to around 70 % of the initial fluorescence intensity in the microgels, the fluorescence fully recovers to 100 %. The solution could even be bleached to roughly 50 % of its initial fluorescence intensity. The recovery curves already show the quickest fluorescence recovery for the solution, followed by the high, medium and lastly low porosity samples. The exact values for the diffusion coefficients were determined using equation (4) and (5). Therefore, the fluorescence recovery and thus the diffusion coefficient correlate with the porosity of the microgels.
